# Isothermal Nucleic Acid Amplification to Detect Infection Caused by Parasites of the Trypanosomatidae Family: A Literature Review and Opinion on the Laboratory to Field Applicability

**DOI:** 10.3390/ijms23147543

**Published:** 2022-07-07

**Authors:** Denis Sereno, Bruno Oury, Anne Geiger, Andrea Vela, Ahmed Karmaoui, Marc Desquesnes

**Affiliations:** 1 Institut de Recherche pour le Développement, Université de Montpellier, UMR INTERTRYP IRD, CIRAD, Parasite Infectiology and Public Health Group, 34032 Montpellier, France; bruno.oury@ird.fr; 2 Centre International de Recherche en Agronomie pour le Développement, Institut de Recherche pour le Développement, Université de Montpellier, UMR INTERTRYP IRD, 34032 Montpellier, France; anne.geiger@ird.fr; 3 One Health Research Group, Facultad de Ciencias de la Salud, Universidad de las Américas-Quito, Calle de los Colimes y Avenida De los Granados, Quito 170513, Ecuador; andrea.vela.chiriboga@udla.edu.ec; 4 Bioactives (Health and Environmental, Epigenetics Team), Faculty of Sciences and Techniques, Errachidia (UMI), Moroccan Center for Culture and Sciences, University Moulay Ismail, Meknes 50000, Morocco; karmaoui.ahmed@gmail.com; 5 CIRAD, UMR INTERTRYP, 31076 Toulouse, France; marc.desquesnes@cirad.fr; 6 INTERTRYP, Université de Montpellier, CIRAD, IRD, 34032 Montpellier, France

**Keywords:** isothermal nucleic acid amplification, diagnostic, trypanosomoses, leishmanioses, chagas disease, point of care

## Abstract

Isothermal amplification of nucleic acids has the potential to be applied in resource-limited areas for the detection of infectious agents, as it does not require complex nucleic purification steps or specific and expensive equipment and reagents to perform the reaction and read the result. Since human and animal infections by pathogens of the Tryponasomatidae family occur mainly in resource-limited areas with scant health infrastructures and personnel, detecting infections by these methodologies would hold great promise. Here, we conduct a narrative review of the literature on the application of isothermal nucleic acid amplification for Trypanosoma and Leishmania infections, which are a scourge for human health and food security. We highlight gaps and propose ways to improve them to translate these powerful technologies into real-world field applications for neglected human and animal diseases caused by Trypanosomatidae.

## 1. Introduction

More than 30 million people are infected by pathogens belonging to the Trypanosomatidae family worldwide, and as many as 100,000 persons die every year from Trypanosoma brucei spp., T. cruzi, or Leishmania spp. infections [1]. Trypanosoma brucei gambiense, T. brucei rhodesiense, and T. cruzi, along with 21 species of Leishmania, are pathogenic causes of human African trypanosomosis (HAT or sleeping sickness), Chagas disease (CD), and cutaneous (CL), mucocutaneous (MCL), or visceral (VL) leishmaniases [2,3,4,5], respectively. Occasional infections with T. evansi, T. lewisi, T. brucei brucei, or T. congolense have been reported in humans, but little is known about the public health importance of these diseases [6]. Trypanosomatid pathogens also infect domestic, feral, and wild animals. Canine visceral leishmaniasis (CVL) is mainly caused by L. infantum infection and occasionally by L. donovani or L. major. Trypanosoma congolense, T. evansi, T. b. brucei, T. vivax, T. simiae, T. suis, and more rarely T. godfreyi affect livestock (cattle, buffalo, camel, horses, sheep, goat, pigs), domestic and wild fauna, causing animal trypanosomoses, while T. equiperdum affects equids [7,8,9], causing a disease called “dourine”.

According to the WHO, all human diseases caused by Trypanosomatid pathogens belong to the neglected tropical diseases, implying that they affect communities, countries, or geographic areas with limited access to healthcare services and with limited financial resources. In addition, since animal trypanosomoses severely affect livestock, especially cattle, they limit agricultural production in potentially the most productive areas of Africa (within the “tsetse belt”) and impact the extensive breeding areas of Latin America (T. vivax and T. evansi), and on the cattle and buffalo farms in Asia (T. evansi). Furthermore, trypanosomosis reduces by 50% the milk, meat, manure production, and draught power, reducing cultivated area and efficiency of resource allocation. Trypanosomosis affects the resources of poor populations, the way farmers manage their livestock, and the number of animals they keep [10]. Furthermore, chemotherapeutic or prophylactic drugs are outdated, and diagnostic tools are limited.

The clinical signs of an infection by pathogenic Trypanosomatid are generally not typical and may be confused with other pathologies. In addition, they may be unapparent in asymptomatic infections and during the chronic phase of the disease, which can last several years in the case of CD. The identification of active infections by these pathogens can be taxing. Detecting the infection requires ad hoc tools with good sensitivity and specificity applicable to the targeted population. As such, “field” applicability might be a significant limitation in developing new and advanced methodologies. At any rate, the term “field” covers a whole range of situations, depending upon the infecting pathogen, the socio-economic condition and environment of the patient, and their access to health care services, whose characteristics are also highly variable, whether for humans or animals. Hence, defining the term “field” is not easy for trypanosomoses and leishmanioses; it has to be defined in the framework of the “point of care” (POC) definition, constrained by the unique epidemiological characteristics of these diseases. The POC is where the service is delivered to the patient, allowing clinicians to document clinical information while interacting with the patient. This definition should exclude animal trypanosomoses, although “POC” is commonly used in animal disease management. The POC for most individuals affected by cutaneous leishmaniasis in the old and new world and possibly persons suffering from CD, HAT, and visceral leishmaniosis have specific characteristics linked to socio-economic and geographic factors. The first approach in diagnosing such infection involves mobile or primary health care services that have to gather the first clues of active infection by trypanosomatid parasites. For AT, the situation is even more radical since the diagnosis must be performed close to the animal; here, the word “field” meaning is actually “pasture”. Therefore, for all these diseases, proper field applicability must ideally target health care services with no dedicated materials (water bath and possibly centrifuge) and often with a lack of trained technicians and nurses.

## 2. Infection by Parasites Belonging to the Trypanosomatidae Family, Challenges for the Detection in Field Conditions

Diseases caused by Trypanosomatidae parasites are prevalent in tropical areas, mainly affecting impoverished communities. Consequently, for confirmatory diagnosis purposes and screening for potential infection, the ideal test should be conducted in peripheral health facilities and mobile labs, at the village level, in zero infrastructure conditions, with non-invasive sample collection.

The detection of these parasites is required on several occasions. First to confirm the clinical diagnosis of a patient, second to detect a risk of transmission in endemic areas through the detection in their arthropod vector, and to detect parasites circulation in their domestic or wild animal hosts.

Leishmanioses include a spectrum of diseases caused by >20 Leishmania species. The detection and identification of the causative agent in patients or animals with symptoms reminiscent of cutaneous, mucocutaneous, and diffuse cutaneous affections involve looking for the parasite in skin biopsy specimens [11]. Identifying the causative Leishmania species to confirm clinical suspicion is essential in the case of mucocutaneous lesions, for persons at risk, for treatment decision of American cutaneous leishmaniasis, and for VL suspicion, the collection of tissue aspirates or biopsy specimens (bone marrow, liver, enlarged lymph nodes, and whole blood) for smears, histopathology, parasite culture, and molecular testing is recommended [11].

Detecting Trypanosoma parasite infections in humans or animals aim to identify a spectrum of parasite species involved in various diseases. The detection of Trypanosoma cruzi infection consists in analyzing clinical, epidemiological, and laboratory data. In patients with suspected acute T. cruzi infection, direct parasitological tests (microhematocrit and direct observation) are recommended. Tests for the presence of parasites may be direct or by multiplication, including hemoculture, xenodiagnosis, and nucleic acid amplification. For the chronic phase, immunological tests are recommended [12]. Therefore, the availability of rapid tests with reasonable specificity and sensitivity, even if their use should be limited to particular circumstances, is required. For human African trypanosomosis, the diagnosis of infection involves analysis of clinical signs and serological tests (screening blood for anti-trypanosomal antibodies). Medical surveys are conducted in villages endemic for the disease using the CATT (card agglutination test for trypanosomosis) [13]. When tests are positive, individuals undergo testing for the parasite presence by microscopy. When positive by microscopy, the disease stage is then determined using lumbar puncture [14] (https://www.who.int/news-room/fact-sheets/detail/trypanosomiasis-human-african-(sleeping-sickness), accessed on 10 May 2022). To detect trypanosomes infecting animals, a recent review depicts the characteristics required to be effective; besides the minimal requirement in terms of sensitivity and specificity, they must detect either antibodies for epidemiological studies or active infection for decision treatment. Seroepidemiological diagnosis must be adapted to large-scale studies, applicable in any regional laboratory, and inexpensive. Active infection detection tests must be affordable, easy to use, and quick to implement, as close as possible to the animals [9]. The ideal test should be conducted in peripheral health facilities and mobile labs, at the village level, in zero infrastructure conditions, with non-invasive sample collection. The diagnostic tests’ characteristics are summarized in TPP (Target Product Profile) available for Gambian HAT, Chagas disease, and cutaneous leishmaniosis. All emphasize the necessity of adapting these tests for “ Health care structures with low complexity laboratories” [15,16,17].

## 3. Isothermal Amplification Methodologies: Application to Pathogenic Trypanosomatids

Transcription-based amplification system (TAS), the first advanced performed on isothermal amplification of DNA and RNA, date back to 1989. After that, several isothermal amplification approaches were designed, requiring various enzymes and primers (Table 1). Four described technologies were applied to trypanosomatidae infection: NASBA, RPA, LAMP, and recently PSR (Table 1).

**Transcription-based amplification system (TAS):** Each cycle of TAS is composed of a DNA synthesis and RNA transcription step. During the cDNA synthesis, short nucleotide sequences recognized by a DNA-dependent RNA polymerase (the PBSs) are positioned on the 3′ side of the target sequence region through an oligonucleotide primer containing two domains. A target-complementary sequence positions the PBS in the desired location before amplification. This oligonucleotide (primer 1) is employed in a primer extension reaction using polymerases. A second primer (2) is used in a similar primer-extension reaction to make a double-stranded PBS-containing cDNA copy of the target sequence. Sequences recognized by the T7 RNA polymerase can be employed. The RNA produced during the first TAS cycle can serve as target sequences for additional TAS cycles [18].

**Self-sustained sequence replication (3SR)** derives from TAS with refinement [19].

**Nucleic acid sequence-based amplification (NASBA):** it required T7 RNA polymerase, RNase H, AMY (avian myeloblastosis virus) reverse transcriptase, and two specific primers. The first primer (about 45 nt) presents an average of 20 bases at the 3′ end that is complementary to the 3′ side of the target sequence. The 5′ end of this primer contains a promoter sequence recognized by T7 RNA polymerase. Primer 2 is about 20 bases in length and is derived from the opposite (5′ direction) side of the target sequence. When adding template RNA, primer 1 anneals to the RNA target sequence. AMY reverse transcriptase extend the 3′ end of primer 1, thereby forming a cDNA copy of the template and generating an RNA:DNA hybrid. RNase H hydrolyses RNA from such RNA–DNA hybrids, leaving a single strand of DNA to which primer 2 anneals. Reverse transcriptase synthesizes the second DNA strand, rendering the promoter region double-stranded. The T7 RNA polymerase transcribes RNA copies from the transcriptionally-active promoter, and each new RNA molecule is available as a reverse transcriptase template. Primer 2 then binds to the template first, and the action of reverse transcriptase extends it, generating an RNA:DNA hybrid. RNase H hydrolyses the RNA strand, primer 1 binds to the resulting single-stranded DNA, and the reverse transcriptase synthesizes DNA, yielding a transcriptionally-active promoter [20].

**Strand DNA Displacement (SDA)** is based on the ability of a restriction endonuclease to nick the unmodified strand of its target DNA, with Escherichia coli DNA polymerase 1 (exo-Klenow) enzyme to extend the 3′-end at the nick and displace the downstream DNA strand. Exponential amplification results from coupling sense and antisense reactions; strands displaced from a sense reaction serve as a target for an antisense reaction and vice versa [21].

**Rolling Circle Amplification (RCA)** is an isothermal method that takes advantage of the hybridization of a primer to a circle DNA. Rolling Circle Amplification uses the bacteriophage Ø29 DNA polymerase enzyme for synthesis at a constant temperature. This method was adapted to two-primer amplification, and these methods are called ramification amplification (RAM), hyperbranched RCA, cascade RCA, or exponential RCA. They have applications in diagnosis and SNP detection [22].

**Loop-Mediated Isothermal Amplification (LAMP)** employs a DNA polymerase and a set of four primers that recognize six distinct sequences on the target DNA. An inner primer with sequences of the sense and antisense strands of the target DNA initiates the reaction. The strand displacement DNA synthesis, primed by an outer primer, releases a single-stranded DNA, which is a template for DNA synthesis primed by the second inner and outer primers that hybridize to the other end of the target. That produces a stem-loop DNA structure. In subsequent LAMP cycling, one inner primer hybridizes to the loop and initiates displacement DNA synthesis, yielding the original stem-loop DNA and a new stem-loop DNA with a stem twice as long [23].

**Multiple Displacement Amplification (MDA)** amplifies the whole genome using multiple primers via the polymerase activity of the bacteriophage Ø29 DNA polymerase [24].

**Exponential amplification reaction (EXPAR)** amplifies DNA in four steps: first, target primes to the trigger sequence of the template, forming a partial double-stranded duplex that initiates the reaction; second, a DNA polymerase ensures the extension by forming an extended double-stranded DNA containing a nicking enzyme recognition site; third, a nicking enzyme then cleaves the upper strand; fourth, DNA polymerase displaces the cleaved trigger by strand displacement to generate additional trigger sequences [25].

**Helicase-Dependent Amplification (HDA)** follows a replication fork mechanism for amplification, which utilizes DNA helicase for unwinding activity. Helicase separates the duplex DNA into single-stranded DNA for the in vitro amplification of target DNA. The DNA extension is done by a DNA polymerase deficient in exonuclease activity. The circular HDA is used for amplifying nucleic acids from a circular DNA template [26].

**Single Primer Isothermal Amplification (SPIA)** requires a single chimeric primer for DNA or RNA amplification. Ribo-SPIA RNA amplification is directed toward the formation of a double-stranded cDNA substrate for subsequent SPIA amplification. The SPIA amplification primer (DNA/RNA chimeric) is complementary to the sequence of the single-stranded 3′ end of the second-strand cDNA in the partial duplex. This chimeric primer comprises a DNA sequence at the 3′ end and an RNA sequence at the 5′ end. DNA amplification is performed by primer extension with a DNA polymerase with strand-displacement activity and by RNase H cleaving the RNA portion of the primer in the RNA/DNA heteroduplex. Cleavage of the 5′ RNA portion of the primer annealed at the priming site clears this site for hybridization of a new primer molecule, which is extended along with the template DNA by DNA polymerase. Strand-displacement DNA synthesis displaces the previous primer extension product away from the template DNA. This cycle of primer binding, extension, displacement, and cleavage causes amplification [27].

**Recombinase Polymerase Amplification (RPA)** is an isothermal method requiring DNA polymerase, recombinase, and DNA-binding proteins; amplification is done at 37–42 °C [28]. Recombinase proteins form complexes with each primer, which scans DNA for homologous sequences. The primers are then inserted at the cognate site by the strand-displacement activity of the recombinase, and single-stranded binding proteins stabilize the displaced DNA chain. The recombinase then disassembles, leaving the 3′-end of the primers accessible to a strand displacing DNA polymerase, which elongates the primer [29]. The cyclic repetition of this process achieves exponential amplification.

**Cross-priming amplification (CPA)** uses multiple cross-linked primers (six to eight primers) to amplify a DNA target sequence at a constant temperature, using Bst DNA polymerase activity **[30]**.

**Primase-based whole genome amplification (T4pWGA)** uses the in vitro reconstitution of the bacteriophage T4 replication machinery to provide a system for rapid and processive isothermal DNA amplification [31].

**Polymerase Spiral Reaction** employs Bst DNA polymerase and a pair of primers. The forward and reverse primer sequences are reversed to each other at their 5′ end, whereas their 3′ end sequences are complementary to their respective target nucleic acid sequences [32].

**Figure 1:**
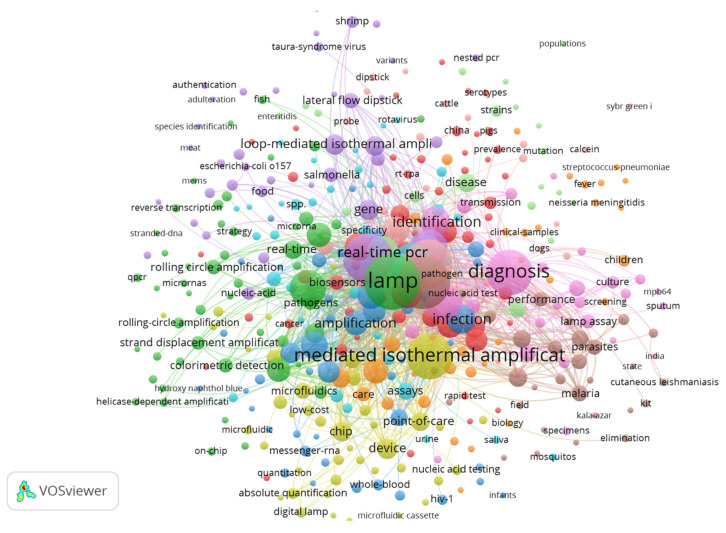
A VOS viewer overlay bibliographic coupling document cluster analysis of the “Isothermal amplification”.

Currently, isothermal amplification methodologies are widely used, and a total of 2526 publications, from 1990 to 4 May 2022, were retrieved from the Web of Science database using “Isothermal nucleic acid amplification”. The trending analysis, performed using a networking software, highlights a series of associated keywords that include: recombinase polymerase amplification, point-of-care diagnostics, point-of-care testing, COVID19, CRISPR, lateral flow assay, low cost, rapid identification, hybridization chain reaction, antibody, platform, miRNA, biosensing, microRNA, gold nanoparticles, colorimetric detection, enzyme-free, nanomaterial and nanotechnology, ultrasensitive detection, label-free detection, and electrochemical biosensor. This analysis also discloses the primary diseases, significant infectious microorganisms, the biological components, characteristics, enzymes, and genetic material associated with the research on isothermal nucleic acid amplification. It points out that isothermal amplification is primarily applied for pathogens (25% Mycobacterium, 15% influenza, 11% Plasmodium, 5% HIV, and 4% hepatitis a). Interestingly, the biological compartment where detection occurs via isothermal amplification includes blood-whole blood 32%, plasma 2%, urine 5%, and cerebrospinal fluid 5%. These compartments are the biological material required to detect parasites belonging to the trypanosomatidae family. A VOSviewer [33] representation of keywords associated with “Isothermal amplification” is given in Figure 1. This figure discloses the association between isothermal amplification and “diagnostic” and “pathogens” keywords. The infection by trypanosomatidae parasites is linked with the “LAMP assay” of amplification and the keywords “field” and “performance”. Nevertheless, Chagas disease, Leishmanioses, human African trypanosomosis, and animal trypanosomosis are under-represented or absent as these methodologies are at an embryonic stage of development for the diagnosis of these diseases.

## 4. Field Detection of Infections Due to Trypanosomatidae: Challenges Overcome by Isothermal DNA Amplification

Does isothermal DNA amplification represent the ideal tool for infection detection? To gather information on such a question, this perfect tool’s characteristics for detecting infection by parasites of the trypanosomatidae family have to be defined. Nevertheless, documents and publications related to “Target product profiles” can help determine them [15,16,17]. In addition to the cost of the amplification procedure, upstream and downstream manipulation necessities must be considered. Upstream manipulation involves sampling; minimal invasive sampling, such as body secretions (e.g., milk, saliva, urine, semen, nasal secretion, lacrimal fluid, earwax, sweat, feces) or appendages (e.g., nail, hair, bristles) are easy and safe to collect and render the diagnosis more convenient since they do not require trained professionals. Information on the interest of such alternatives for detection was systematically reviewed for infection caused by Trypanosomes and Leishmania [34]. Supply chain (transport and storage) and sample preparation that include minimal or complete nucleic acid preparation are limitations to test field deployment. For the reaction by itself, affordability not only depends on the number of enzymes and primers required to carry it out, but also in the need for a costly dedicated apparatus and on the storage requirement for samples and enzymes (Room temperature, 4 °C, minus 20 or 80 °C). Downstream applications required to reveal the test (Agarose gel, fluorescence, fluorimetry, oligo chromatography, turbidimetry, etc.) also limits the field applicability. Consequently, for diagnosis, confirmatory purposes, and screening for potential infection, the ideal test must be conducted in peripheral health facilities and mobile labs at the village level in zero infrastructure conditions with non-invasive sample collection. Here, we collect data on isothermal DNA/RNA amplification technology whose information is available on trypanosomatidae parasites and critically assess their suitability in the light of ideal requirements: to be conducted in peripheral health facilities (mobile labs at the village level and zero infrastructure conditions) with samples non-invasively collected.

**Figure 2:**
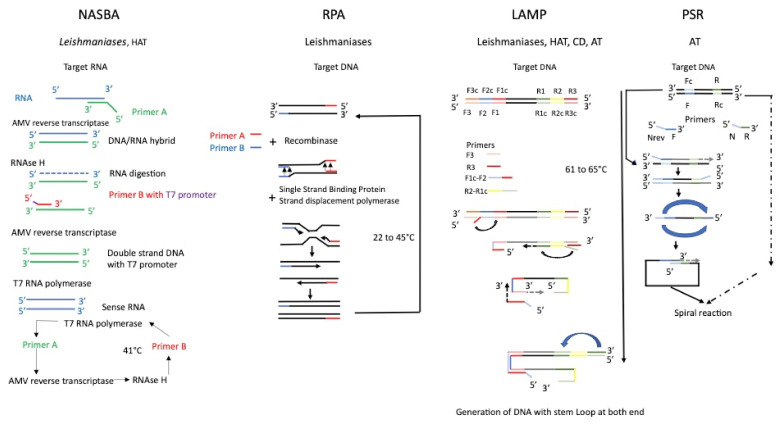
Schematic representation of isothermal amplification tested on Trypanosomatidae parasites. HAT—human African trypanosomosis; CD—Chagas disease, AT—animal trypanosomosis.

Research on isothermal nucleic amplification on Pubmed reveals that a limited number of technologies are tested on pathogenic trypanosomatidae, including NASBA, RPA, LAMP, and PSR; a synthetic reaction scheme of these isothermal methods of nucleic acid amplification is given in Figure 2.

From the collected research papers, criteria to translate isothermal tests into field settings were extracted; sources of biological material, nucleic acid preparation, methods of isothermal amplification, and detection of positive/negative reaction (Table 2). In this table, we do not quote data on sensitivity that varies from 45% to 100% or specificity, almost always superior to 90%; heterogeneity of the methodology related to sample origins (vertebrate and invertebrate hosts, biological source of samples, etc.), processing of samples (extraction of DNA with the various kit, other methodologies…), type of isothermal amplification and their targets, and downstream methods used to visualize results (Eyes, UV, colorimetric, agarose gel electrophoresis, oligo chromatography, fluorimetry, dedicated device…) are reviewed for Leishmania infection [35,36]. We instead analyze the steady-state level of tests to be translated into the field medical setting for visceral and cutaneous leishmaniasis, Chagas disease, and human or animal trypanosomosis. In addition, data on insect vectors were also collected. We then calculated and aggregated a synthetic score of “field applicability”.

For RPA, the implementation of mobile suitcase laboratories, needed for separate nucleic acid extraction and detection workspaces, has pushed towards its application in point-of-care settings. These suitcases have undertaken a phase 2 multi-country study for *L. donovani* detection in patients suffering VL, CL, or PKDL [101]. However, they have not been tested in HAT, CD, or AT settings. Furthermore, no methodology was developed for detecting *T. brucei* spp., *T. cruzi*, or *Trypanosomes* responsible for animal trypanosomoses. Nevertheless, the high cost of material acquisition and disposable kits for DNA extraction, amplification, and detection would limit their use in most settings. Therefore, they have not been considered for critical analysis.

The synthetic score of field applicability corresponds to the sum of the five criteria required to perform a complete test in a “field” medical setting (Table 2).

- The first criteria correspond to the biological sample origin: highly invasive (blood, CSF, bone marrow, etc.) (3), moderately invasive (drop blood, punch, etc.) (2), minimally invasive (urine, saliva, swab, etc.) (1).
- The second to the processing of samples before isothermal amplification: DNA extraction by commercial kit, phenol-chloroform, etc. (3), simple extraction protocol (chelex and simplified published protocols) (2), no extraction (crude), and minimal lysis procedure (boiling, detergent, etc.) (1).
- The third is the Isothermal amplification methodology: three enzymes (3), two enzymes (2), one enzyme (1). An additional handicap (one point is given to methodologies needing more than two primers).
- The mode of detection: complex (fluorimetry, oligo chromatography, Agarose gel, microfluidic, etc.) (3), low complexity (UV light, turbidimetry, etc.) (2), simple (naked eyes, with dye or not, other) (1).

### 4.1. Supply Chain and Storage

Besides criteria linked to the biological process, tests’ supply and storage chain can be highly limiting for neglected tropical that primarily affects populations far from equipped medical settings. Interestingly, good stability after 1–15 days of storage at −20 °C, 2.5 °C, or 37 °C of LAMP reactive products is documented for assays with FTA blotted and crude ex in vivo reconstituted hemolyzed blood containing purified genomic DNA of *T. brucei brucei*. This adds information on the suitability of such tests for in-field detection of Trypanosoma parasites but remains to be evaluated in field conditions of infection [102]. Alternatively, the industrial implementation of the LAMP kit allowed the development of lyophilized reaction products overcoming problems of supply and storage [81].

### 4.2. Sampling and Samples Processing

The reliability of some minimally invasively acquired biological samples to track trypanosomatidae pathogens has never been thoroughly investigated; only preliminary data are available for some of them [34]. Nevertheless, such biological material is paramount for the field deployment of diagnostic tests.

Implementing a Nucleic acid-extraction device (PrintrLab), constructed by repurposing a low-cost three-dimensional printer to provide purified nucleic acids and LAMP incubation step for point-of-care molecular applications was successful in detecting *T. cruzi* DNA in the various clinical presentation of Chagas disease [103]. However, this process corresponds to “acceptable operational characteristics” of the target product profile implemented for Chagas disease health care facilities [16] but not for low complexity field laboratories frequently encountered for diagnosing patients.

### 4.3. Isothermal Amplification

From the short literature survey, isothermal amplification procedures tested on pathogenic trypanosomatids of medical or veterinary interest demonstrated a robust amplification capacity. The LAMP methodology is the most employed one.

### 4.4. Result Acquisition and Interpretation

Lateral flow to detect antigens or antibodies was adapted to detect nucleic acid amplification products. Lateral flow tests based on Au-nanoparticle aggregation tests are used as POC devices and are easy to read with the naked eye, but the risk of cross-contamination with amplimers is still present. Paper-based diagnosis devices using molecular fluorescent labels increase the cost, add disposal problems (most of them are toxic), and present instability problems (photobleaching). To limit all these risks and notably the cross-contamination one, LAMP amplification was integrated into a contactless/label-free conductivity detection system [104]. The field applicability of this system remains to be determined. Turbidity is the cheapest way to detect positive reactions, but various simple methodologies are available. SYB Green, often used to reveal LAMP reaction, is costly, and its intercalating activity inhibits multimerization with *Bst* polymerases, impacting the sensitivity [105]. Malachite green represents an alternative tested and validated on LAMP *Leishmania* detection but is photodegradable [69,106]. Other options included Molybdate blue, which traces the amount of inorganic phosphate released during the reaction [107], or naphthol blue [108].

Some members of the CRISPR family (Casl2, Casl3, and Cas14) present guide RNA (gRNA) sequence-specific recognition and endonuclease activity, also called CRISPR RNA (crRNA), and target-activated *trans*-cleavage activity. These properties have permitted the development of a range of new methods for nucleic acid detection. These methodologies present many advantages since they increase sensitivity by the fluorescent read-out and specificity conferred by the guide crDNA or crRNA. For example, CRISPR-Cas 13 (SHERLOCK) [109] or CRISPR-Cas12 (HOLMES: a one-Hour Low-cost Multipurpose highly Efficient System) [110] were tested with success on CL and HAT clinical samples [111,112]. These methodologies detect Nucleic acid but require an initial step for amplifying the genetic material (RNA or DNA) by isothermal or classical PCR methodologies. The initial nucleic acid amplification step can be considered a limitation for field deployment in resource-limited conditions encountered by patients or animals suffering trypanosomatidae infections.

### 4.5. Specificity and Sensitivity

To date, available isothermal methods focused on the detection of Trypanosomes belonging to the Trypanozoon subgenus, *Leishmania*, or the subgenus Viannia (see Table 2). Additionally, a limited number of tests with species specificity are available: *T. b. rhodesiense* (SRA), *T. b. gambiense* (TgsGP), *T evansi* type A (ROTAT 1.2 VSG, ISG), *T. congolense* (CON2 18s rDNA), and *T. vivax* (Sat DNA) (see Table 2). For *T. cruzi*, specific detection of DNA is available, as for *T. rangeli*, which is not of medical importance. Concerning *Leishmania*, knowing the vast diversity of human and animal pathogenic species (at least 21 over more than 50 described), only *L. donovani*, *L. infantum*, *L. amazonensis L. major*, and *L. tropica* can be identified at the species level via LAMP isothermal amplification. Therefore, future research might involve the set-up of tests allowing the identification at the species level to guide chemotherapy and survey disease epidemiology.

Among isothermal methods tested on infections caused by parasites of the Trypanosomatidae family, NASBA required complex reaction components and the use of three enzymes. In addition, the need for a particular device to detect the amplification product required is not easy nor cheap—all impact the cost-effectiveness of this methodology and its field applicability. In addition, the design of primers is not easy, limiting their future application. The RPA reaction also requires multiple enzymes in the amplification system and a probe to detect the reaction, which increases its cost. RPA primers and probes can amplify and detect target genes containing 5–9 mismatches, which might impact the specificity and the sensitivity as well [113]. For LAMP reaction, the cumbersome primer design that needs to be software-assisted and the final product complexity are limitations to LAMP implementation.

Nevertheless, because of its sensitivity, specificity, and ease of detection, the LAMP methodology is the most field applicable currently available. Regardless PSR, which did not require a complex design of primers, was evaluated only on *T. evansi* [95]. The design of new species primers or the use of already characterized species-specific primers might accelerate the path of PSR application.

Besides methodologies, several strategies to enhance sensitivity and specificity are available. They include the adjunction of enzymes (helicases, recombinases, endonucleases) to eliminate the denaturation step and decrease the nonspecific interaction, lowering the melting temperature. Nevertheless, these increase the complexity and cost of the reaction. Alternatively, adjunction of ionic liquid or Betaine, Proline, Trehalose, etc., to stabilize enzymes and decrease the melting temperature of the reaction are alternatives to improve sensitivity and specificity of the isothermal reaction [114] and reviewed by [115]. The CRISPR-Cas-based nucleic acid detection methodologies can also improve sensitivity and specificity but with limitations for their field deployment (see Chapter 4.4). It has been tested with RPA to detect Trypanosomes responsible for HAT [112].

### 4.6. Amplimer Cross-Contamination

Amplimer cross-contamination is a tremendous problem impacting detection accuracy via nucleic acid amplification methodologies. However, minimal manipulation of biological samples and minimum pipetting operations would limit such amplimer contamination.

### 4.7. Detection of Trypanosomatids Parasites in their Arthropod Vector

Detection of trypanosomatids in their insect vector is required on multiple occasions: the xenodiagnosis, the survey of transmission risk, and the search for potential vectors. Isothermal amplifications are suitable for all these eventualities. Notably, data on mechanical vectors are lacking even if these technologies should be of paramount importance to search for Trypanosomes nucleic acid in such insect vectors, knowing their sensitivity and ease of handling in field conditions. Mass screening dedicated to field survey or vector incrimination has been readily tested on sandflies with minimal processing of samples (crushing and boiling the whole sandfly) and a simple method of detection (Malachite green) (see Table 2 for references). For xenodiagnosis, methodologies needing tsetse dissection and DNA extraction protocol have been tested successfully (see Table 2 for references). Concerning *Triatoma* spp. or *Rhodnius* spp., the procedure used needs insect dissection and DNA extraction, limiting its interest (Table 2).

## 5. Conclusions and Perspectives

Typanosomoses and Leishmanioses affect communities, countries, or geographic areas with limited or no access to healthcare services and limited financial resources. Animal trypanosomosis strongly affects cattle, thus impacting farmer communities’ food security and animal trading. Therefore, the detection of infection requires methodologies adapted to such conditions (restricted access to technologies) but must also ideally require a limited intervention of specialized and highly skilled personnel (nurse, lab technician, etc.). The ideal field implementation and application must comply with affordable cost, little technological requirement, easiness in storage, manipulation, and read-out, and minimal manipulation to avoid amplimer contamination. Currently, field translation of isothermal amplification methodologies to neglected tropical diseases caused by Trypanosomatid pathogens is at its embryonic stages. Efforts must be made to (i) address the diversity of pathogens causing these diseases, (ii) identify minimally, non-invasive methods of sampling, (iii) avoid DNA/RNA purification processes, and (iv) detect positive reactions without needing technology.

## Figures and Tables

**Table 1:** Principal isothermal nucleic acid amplification methodologies.

Name	Year	Den-T (°C)	Nb-Enz	Primers	Nucleic Acid	Ref.
Transcription-based amplification system (TAS)	1989	Yes—Multiple	2 Reverse Transcriptasease T7 RNA polymerase	2	RNA 150 nt	[18]
Self-sustained sequence replication (3SR)	1990	Yes—Multiple from 37 to 61	3 AMV RTase RNase H, T7 RNA pol		RNA DNA 200 nt	[19]
Nucleic acid sequence-based amplification (NASBA) *	1991	Yes—41	2–3 AMV RTase RNase H T7 RNA pol	2	RNA DNA	[20]
Strand DNA displacement amplification (SDA)	1992	Yes—37	2 Rest Enz(s) DNA pol (Kleenow)	2–4	RNA DNA	[21]
Rolling circle amplification (RCA) Ramification Amplification Method (RAM) hyperbranched RCA (hRCA) Cascade RCA (cRCA) Exponential (eRCA)	1998	No	1 DNA pol		RNA CircDNA	[22]
Loop-mediated isothermal amplification (LAMP)	2000	Yes—60–65	1 DNA pol	4–6	RNA DNA	[23]
Multiple displacement amplification (MDA)	2002	No—30–37	1 DNA polymerase	Random primers	DNA >10 kb Genome	[24]
Exponential amplification reaction (EXPAR)	2003	Yes—60	2 DNA polymerase Nicking enzyme	4	DNA	[25]
Helicase-dependent amplification (HAD) Circular helicase-dependent amplification (cHDA)	2004	No—37–65	3 Helicase DNA pol SS-DNA-BP	2	DNA	[26]
Single primer isothermal amplification (SPIA and Ribo SPIA)	2005	60	3 Rev Tase RNAse H DNA pol	1	RNA-DNA	[27]
Recombinase polymerase assay (RPA)	2006	37–42	3 recombinase strand-displacing polymerase single-stranded DNA binding protein	2	DNA	[28]
Cross-priming amplification (CPA)	2009	65	1 *Bst* DNA pol	6–8	DNA	[30]
Primase-based whole genome amplification (T4pWGA)	2010	37	2 T7gp4 primase DNA polymerase	0	DNA circDNA	[31]
Polymerase spiral reaction (PSR)	2015	No—60–65	1 *Bst* DNA pol	2	RNA DNA	[32]

**Table 2:** Methodologies needed to process biological samples reported in the literature involving isothermal amplification of nucleic acids: from biological sampling to results acquisition and their field translation score.

Dis	Pathogens	Hosts	Targets	Upstream		Reaction	Downstream	Refs.	Sc
				BiolS ^&^	Purif *		Vis/Quant		
VL	*L. donovani*	H	18s rDNA	Blood	Yes	NASBA	Oligochromatography	[37,38]	12
VL	*L. donovani*	H	ITS1 Ldonovani repeated sequence	Blood drop	Yes	LAMP	NE Fluorimetry Agarose gel	[39]	11
VL	*Leishmania* *L. donovani*	H	kDNA 18s rDNA	Blood	Boil and spin	LAMP	Fluorescence	[40,41]	10
VL	*L. infantum*	H	kDNA	Bone marrow	Yes	LAMP	NE	[42]	10
VL	*Leishmania*	H	18s rDNA	Blood buffy-coat	Yes	LAMP	NE	[43]	10
VL	*Leishmania* *L. donovai*	H	18s rDNA kDNA	Blood Bone marrow	Boil and spin	LAMP	Fluorimetry	[44]	10
VL	*L. infantum*	H	K26 hydrophilic Ag (AF131228)	Blood	Yes	LAMP	NE (turbidity)	[45]	10
VL	*Leishmania* *L. donovani*	H	18s rDNA kDNA	Blood buffy-coat	Boil and spin Yes	LAMP	NE Blue Led	[46]	8
VL	*Leishmania*	H	18s rDNA	Blood	Boil and spin Yes	LAMP	NE	[47]	8
VL	*L. donovani*	H	kDNA	Blood	Yes	LAMP	NE Agarose gel	[48]	10
VL	*L. donovani*	H	kDNA	Blood	Yes	Q-RPA	Fluorescence (real-time)	[49]	11
VL	*L. donovani* *Others*	H	kDNA	Blood	Yes	RPA	Fluorescence	[50]	12
VL	*L. donovani*	H	kDNA	Blood/buffy-coat	Yes	LAMP	NE Agarose gel	[51]	10
VL PKDL	*Leishmania* spp.	H	18s rDNA	Skin biopsy Blood	Yes	NASBA	Oligochromatography	[52]	11
VL PKDL	*L. donovani*	*H*	kDNA	Blood Bone marrow Skin biopsy	Yes	LAMP	NE	[53]	10
PKDL	*L. donovani*	H	kDNA	Skin biopsy	Boil and spin Yes	RPA Q-RPA	Fluorescence (real-time)	[54]	10
PKDL	*L. donovani*	H	kDNA	Punch tissue	Yes	LAMP	Fluorimetry	[55]	10
VL PKDL	*L. donovani*	H	kDNA	Blood Skin biopsy	Yes	LAMP	Fluorescence (device)	[56]	12
VL CL	*Leishmania*	H (HIV+)	18s RNA	Blood/buffy-coat	Yes	LAMP	NE Fluorescence Agarose gel	[57]	10
VL CL	*Leishmania* *L. donovani*	H	18s rRNA	Blood Skin biopsy	Yes	RT LAMP	Fluorescence NE	[58]	11
VL CL	*Leishmania*	H	18s rDNA	Blood Skin biopsies	Yes	LAMP	NE Fluorimetry (RT)	[59]	10
CL ML	*L. amazonensis* *L. braziliensis*	H	18s rDNA	Skin biopsy Blood	Yes	NASBA	Oligochromatography	[37]	11
CL	*Leishmania*	H	18s rDNA	Scrap FTA	No	LAMP	NE (Malachite green)	[60]	6
CL	*Leishmania*	H	18s rRNA	Skin biopsy	Yes	NASBA	Chemiluminescence	[61,62]	11
	*L. major* *L. tropica*	H	CPB	Lesion aspirate	Yes	LAMP	NE Agarose gel	[63]	9
CL	*Leishmania (Viannia)*	H	NA	Lesion scrap	Yes	RPA	Lateral flow fluo	[64]	10
CL	*Leishmania (Viannia)*	H	kDNA	Ulcer juice FTA	Yes	RPA	Lateral flow	[65]	10
CL	*L. donovani*	H	kDNA	Punch biopsy	Yes	RPA	Fluorescence	[66]	10
CL	*Leishmania (Viannia)*	H	kDNA	Swab-FTA	Boil	RPA	Lateral flow	[67]	8
CL	*Leishmania*	*P. panamensis* *M. cayennensis* *L. gomezi*	18s rDNA	Sandfly	Yes	LAMP	NE Agarose gel	[68]	
SF	*Leishmania*	*L. ayacuchensis*	18s rDNA	Whole SF	Simplified	LAMP	NE (Malachite green)	[69]	
SF	*Leishmania*	*P. papatasi* *P. alexandri* *S. sintoni* *S. baghdadis*	18s rDNA	Thorax and abdomen	Yes	LAMP	Fluorescence (EP)	[70]	
SF	*Leishmania* *L. martiniquensis*	*S. gemea*	18s rDNA	Whole SF	Yes	LAMP	NE (Malachite green)	[71]	
SF	*L. donovani*	*P. argentipes*	kDNA	Whole SF	Yes	RPA	Fluorescence	[72]	
Canl	*L. infantum*	Dogs	kDNA	Blood-FTA Mucosal scraping-FTA	Yes	RPA	Lateral flow	[73]	10
CanL	*L. infantum*	Dogs	kDNA	Conjunctival swab	Boil	LAMP	NE Turbidimeter Agarose gel	[74]	6
CanL	*L. infantum*	Dogs	CPB	Blood	Yes	LAMP	NE Turbidimeter	[75]	10
CD	*T. cruzi*	H Chronic and acute	Satellite DNA	Blood	Yes	LAMP	NE Fluorimetry	[76]	10
CD	*T. cruzi*	H	Satellite DNA	Blood-EDTA Drop blood spot CSF	Yes	LAMP	NE	[77]	10
CD	*T. cruzi* *T. rangeli*	*R. pallescens* *T. infestans*	18s rDNA SnoRNA	Digestive tract	Yes	LAMP	NE	[78]	
HAT	*T. brucei spp.*	H	18s rDNA	Blood-CSF	Yes	NASBA	Oligochromatography	[79,80]	14
HAT	*Trypanozoon* *T. b. rhodesiense*	H	RIME SRA	Blood	Yes	LAMP	Fluorescence	[81]	11
HAT	*Trypanozoon*	H	RIME	Blood/buffy-coat CSF	Boil Supernatant	LAMP	Agarose gel Fluorimetry	[82]	10
HAT	*Trypanozoon* *T. b. rhodesiensis*	H	RIME SRA	Blood-FTA CSF	Yes	LAMP	Fluorescence transilluminator	[83]	11
HAT	*Trypanozoon* *T. b. rhodesiensis*	H	RIME SRA	Blood	Yes	LAMP	NE	[84]	10
HAT	*T. b. gambiense*	H	TgsGP	Blood-CSF	Yes	LAMP	NE UV SYB Green	[85]	10
HAT	*Trypanozoon*	*G. m. morsitans* *G. pallidipes*	RIME	Whole tsetse Midgut	Yes	LAMP	NE Agarose gel	[86]	
HAT	*Trypanozoon*	*G. m. morsitans*	RIME	Whole tsetse	Yes	LAMP	In kit (Led 500 nm) NE	[87]	
HAT	*Trypanozoon*	*G. f. fuscipes*	RIME	Salivary gland	Yes Chelex	LAMP	Fluorescence	[88]	
AT	*T. vivax*	Cattle	Satellite DNA	Blood	Yes	LAMP	Agarose gel	[89]	12
AT	*T. evansi* B	Camel	VSG gene JN 2118Hu	Blood	Yes	LAMP	Agarose gel NE-SYBgreen Lateral flow Dipstick	[90]	11
AT	*T. evansi*	Buffalo	RoTat 1.2 VSG	Blood	Yes	LAMP	NE- acidic molybdate and potassium antimonyl tartrate solution Agarose gel	[91]	10
AT	*T. evansi*	Domestic animals	RoTat1.2 VSG	Blood	Yes	LAMP	NE Agarose gel	[92]	10
AT	*T. evansi*	Pig	PFR *A1*	Blood	Yes	LAMP	NA	[93]	10
AT	*T. evansi*	Camel	RoTat1.2 VSG	Blood	Detergent Tx100	LAMP	UV	[94]	9
AT	*T. evansi*	Equine	Invariable surface glycoprotein (ISG)	Blood	Yes	PSR	NE	[95]	9
AT	*Trypanozoon* *T. brucei spp.*	Equids	RIME	Blood CSF	Boil	LAMP	NE Fluorimetry	[96]	8
AT	*Trypanozoon* *T. b. rhodesiense* *T. congolense*	Dogs	RIME SRA CON2 18S rRNA	Blood-FTA	Yes	LAMP	Fluorescence (EP) transillumination	[97,98]	11
AT	*Trypanosoma spp.*	Cattle	PFR	Blood	Yes	LAMP	Turbidimeter	[99]	11
AT	*T. vivax*	Bovine Tsetse	Sat DNA T. vivax	Blood/buffy-coat Whole tsetse	Yes	LAMP	Fluorimetry Agarose gel	[100]	12

Dis Diseases: CL, ML, VL, and PKDL are cutaneous, mucosal, visceral, and post-kala-azar dermal leishmanioses, respectively. ^&^ Biological samples used to perform the test. * Yes, points to the use of the commercially available kit, Phenol chloroform methods, custom complex protocols, such as the Boom or Chelex methodologies, etc. Tx100—Triton X100; Sc—score of field applicability highest 4 to lowest field translation capability 12; NE—Naked eyes; RIME—repetitive insertion mobile elements; SRA—serum-resistant antigen; PFR A1—paraflagellar rod protein A1; TgsGP—*T. b. gambiense*-specific glycoprotein; RoTaT1.2—Rode Trypanozoon Antigenic Type 1.2; CPB—Cystein protease B. A black border frames top-ranking scores of field applicability. Shaded grey column indicate isothermal amplification performed on insect vectors.

## Data Availability

Not applicable.
